# Optical and thermal properties of edible coatings for application in solar drying

**DOI:** 10.1038/s41598-021-88901-5

**Published:** 2021-05-12

**Authors:** A. López-Ortiz, I. Y. Pacheco Pineda, L. L. Méndez-Lagunas, A. Balbuena Ortega, Laura Guerrero Martínez, J. P. Pérez-Orozco, J. A. del Río, P. K. Nair

**Affiliations:** 1 Instituto de Energías Renovables, Universidad Nacional Autónoma de México, Morelos, 62580 Mexico; 2 Instituto Tecnológico de Zacatepec, Departamento de Ingeniería Química y Bioquímica, Tecnológico Nacional de México, Morelos, 62780 Mexico; 3 Instituto Politécnico Nacional, Centro Interdisciplinario de Investigación para el Desarrollo Integral Regional (CIIDIR), Oaxaca, 71230 Mexico

**Keywords:** Energy science and technology, Carbohydrates, Polysaccharides, Chemical modification

## Abstract

Solar drying is a sustainable process that may impact the quality of dried food. This is because, pigments contained in food are sensitive to sunlight, and exposure to ultraviolet radiation can affect them. We applied biopolymer-based coatings on strawberry, from hydro-colloidal solutions of *Opuntia ficus indica*-mucilage, fenugreek, xanthan gum, gum Arabic, and guar gum to evaluate their potential use as UV filters for solar drying of food. Thermal properties and the optical transmittance, absorbance and reflectance of the coatings were measured to assess their influence on food-sunlight interaction. During the drying experiments, the moisture content, total anthocyanins (TA), and total phenolic compounds (TPC) were measured. Optical and thermal properties are influenced by the biopolymer-based coatings. Also, the optical properties are influenced by the coating thickness. The differences in optical and thermal properties influence the drying process. Differences exist in the drying rate for strawberry slices with coating, compared with those without the coatings. In general, the TA and TPC content in the product are better preserved under solar drying than in control experiments done in a drying oven. A partial transmittance of solar UV radiation is recommended to obtain increased TA and TPC contents in the dried product.

## Introduction

Open sun drying is an old food preservation method used since 8000 BC^1,2^ and it remains widely used in different countries. This conservation technique has shown disadvantages such as a fungal attack, unexpected rain, adverse weather conditions, contamination due to birds droppings, insects, and rodents^3^. The materials subjected to dehydration directly under the sun receive solar radiant energy as well as its ultraviolet radiation (UV) content. Hence, during the solar drying, in addition to the reactions, which may lead to thermal degradation such as oxidation, enzymatic and non-enzymatic darkening, the UV radiation impacts the food quality, mainly arising from photosensitive bioactive compounds. Furthermore, in solar drying, the direct exposure of food to sunlight may degrade its quality: loss of its natural color, destruction of vitamins and an overall degradation of nutritional value, arising from ultraviolet rays^4,5^. Terrestrial solar radiation spectrum includes three relevant bands: ultraviolet, visible, and infrared (IR). IR radiation provides 49.4%, visible light 42.3%, while UV radiation makes up 8.2% of its energy content in most drying sites^6^. Each of these bands has a different impact on the living organisms, as well as on the food during its production, harvest and drying.

The infrared content of the solar radiation, located within the wavelength ($\lambda$) interval of $700<\lambda <1000$ nm, heats the sample, but it does not contribute substantially to the chemical reactions. Visible light ($400<\lambda <700$ nm) influences many substances of biological importance that have a selective chemical response in the visible part of the solar spectrum, causing transformations of the molecular structure with different biological functions such as photosynthesis. UV radiation ($\lambda <400$ nm) is absorbed by electrons of the atoms and molecules and it can change the molecular structure and produce significant chemical changes. In the UV radiation that reaches the Earth’s surface, only a small fraction of it in the UV spectral range of 280–320 nm (UV-B) appears to affect the photosensitive phytochemicals present in food^7^.

The anthocyanins are among the most important phytochemicals found in berries like strawberries, raspberries, blueberries, blackberries, red currants, whitecurrants and blackcurrants. These compounds confer their red to the dark-blue color and are located in the vacuoles of almost all types of cells in the epidermal, terrestrial and vascular tissues of all the vegetative organs. They are highly sensitive to light, UV-B radiation, and extreme temperatures^8,9^.

Numerous studies of berry drying have been reported; however, the effect of direct solar drying on bioactive berry compounds is not widely known. Mulokozi and Svanberg^10^ evaluated the effect of open sun drying and solar cabinet drying on $\beta$-carotene and vitamin C, they found major retention of both these components in solar drying due to a reduction in sunlight incidence. Meanwhile El-Beltagy et al.^11^ found a major loss of ascorbic acid with an increase in the exposed surface area of the strawberries to sunlight. Both of these studies included the influence of solar thermal effect. However, the prevention of detrimental effects on the chemical composition of food during solar drying requires more attention.

Several techniques protect bioactive compounds in the food during the dying by reducing their thermal degradation. In particular, hydrocolloids from polysaccharides are used. Some examples are xanthan gum^12^, guar gum^13^, gum Arabic^14^, Opuntia mucilage^15,16^ and fenugreek mucilage^17^. Their role include as: thickening and gelling agents of aqueous solutions, stabilizers of foams, emulsions, and dispersion, crystal growth inhibitors, encapsulating agents, and producers of edible films^18^. These hydrocolloids also provide fine texture, sensory and flavor properties, chemical compositions and bio-functionality properties in the dried product.

Edible films and coatings have been used on fresh food products to block oxygen, microorganisms, and moisture or as solute movement regulator to achieve extended shelf life or by regulating respiration rates and thereby inhibiting weight loss during their storage^14,19^. Also, edible coatings have been used as protective and preservative barriers for raw vegetables against sunlight damage^20^ or as treatment during storage under light pulses^21^. Nevertheless, the use of polymers as a solar filter during solar drying is a novel application.

The gum Arabic, xanthan gum, and guar gum has been usually used as an additive in food products and as a coating in raw food products with acceptance of their quality from expert panelists^22–24^. Mucilage of *Opuntia ficus indica* has also been used as a coating in raw fruits with a positive sensory acceptance^25–27^. Fenugreek coating is usually perceived to cause a bitter flavor. Nevertheless, it is consumed due to their beneficial properties leading to a positive acceptability as an additive in food products^28^ The main components of the opuntia mucilage are 24.6–42% of arabinose; 21–40.1% of galactose; 8–12.7% of galacturonic acid; 7–13.1% of rhamnose and 22–22.2% of xylose^16,29^. The main components of the fenugreek mucilage are mannose:galactose (1:1)^30^. During drying, a protective effect of edible coating to reduce the loss of vitamin C, trans-$\alpha$-carotene and trans-$\beta$-carotene in pineapple and pumpkin has been observed^31^. Also, starch coatings, pectin, and biopolymers have been evaluated as a protector agent to prevent color deterioration during drying^32–34^. Another example is the use of *Opuntia* mucilage coating, extracted from the cladodes (modified stems) of *Opuntia ficus indica*, on strawberries during storage and on a type of banana during drying with good results in preserving color by inhibiting or reducing polyphenol oxidase (PPO) activity^35,36^. The barrier generated by the mucilage can be applied to prevent color deterioration in the strawberries, since the characteristic red color is related to anthocyanins. Thus, the films’ use may increase the quality of the food, but they will limit the mass and heat transfers due to the generated barrier.

Heat transfer from the air to the food is desirable in the drying process. Consequently, covering films has a dominant role in the transport phenomena in the drying process. The study of composition, rheological, physical, physicochemical, functional, microstructural, swelling properties and transport properties of edible gums has been widely and well studied^37–45^. On the other hand, energy absorption and heat transfer are essential factors to study the radiation’s effects on food during the solar drying process. Nevertheless, the interaction between the infrared to UV radiation with edible coatings is not well described. A controlled study under lab conditions with a controlled radiation spectrum would give insight to how these edible films influence the heat transfer during drying of coated berries and its relation with any loss of properties of anthocyanins. Furthermore, this study also will provide information about the interaction between radiation and coatings.

The potential use of hydrocolloids as UV filters during solar drying has not been previously studied, we analyze the possibility of this use. Moreover, here we present some relations between anthocyanins and optical properties of edible films. Therefore, this work aims to evaluate this aspect of hydrocolloids (mucilage extracts of plants and natural gums) as edible coating with special attention to their UV filtering properties, thermal behavior, drying kinetics and subsequent effect on the total anthocyanins (TA) and total phenolic compounds (TPC), as seen in strawberry drying.

## Results and discussion

In this section, we discuss the results obtained from solar drier and oven drier experiments. We compare the results obtained with coated strawberries using different edible films described by the following nomenclature M: *Opuntia ficus-indica* mucilage, F: fenugreek mucilage, X: xhantan gum, G: guar gum, A: gum Arabic and Ref: un-coated samples, respectively.

### Optical properties

We evaluate the optical parameters of natural coatings in a wavelength range from 190 to 2500 nm. The average thickness of the different coatings produced from 4 $\upmu$L and 8 $\upmu$L of hydrocolloids solutions (Figure S4) is reported in Table 1. We found significant differences ($\alpha$ = 0.5) in average thickness due treatments. Note the higher thickness of the fenugreek mucilage (F) coating (11–13.9 $\upmu$L) and the smaller thickness of the guar gum (G) coatings (0.16–0.23 $\upmu$L).

**Table 1:** Edible film thickness.

Edible film	Thickness average (nm)	
	$4 \upmu L$	$8 \upmu L$
M	$3190.9 \pm 250.6$	$4209.7 \pm 139.5$
F	$11,127 \pm 1118$	$13,909 \pm 2482$
A	$174.0 \pm 42.8$	$219.7 \pm 30.9$
X	$815.4\pm 26.7$	$2039.7\pm 94.35$
G	$161.3 \pm 87.13$	$231.1 \pm 171.4$

**Figure 1:**
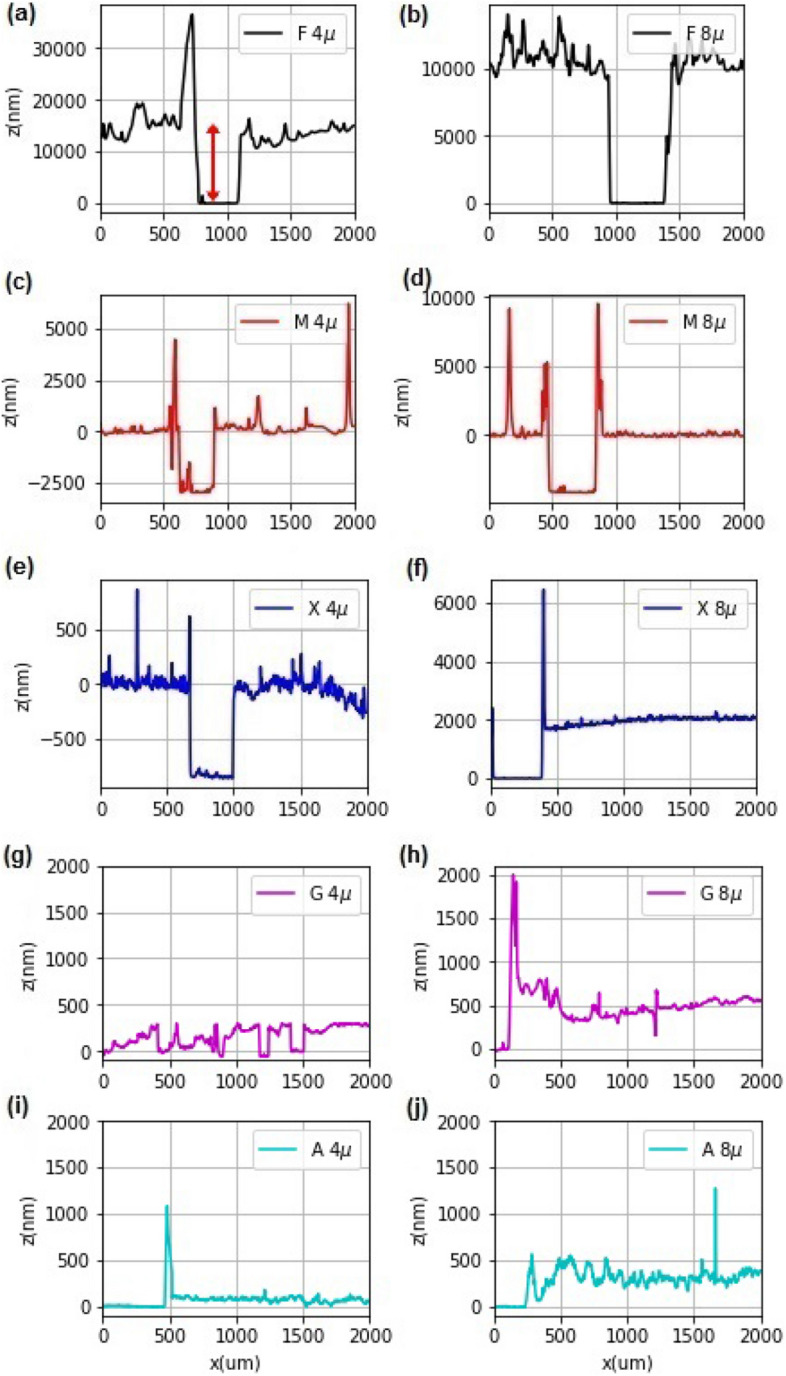
Surface roughness of the dried solution-cast coatings recorded along a 2 mm scan over the surface, involving a step in some cases.

All of the dried coatings (Fig. 1a–f) are rough, all along the 2 mm span for the scan, and thus constitute an isotropic roughness. However, Fig. 1g–j have more random thickness variation pattern on a vertical scale. These differences may lead to differences in their optical properties, and hence influencing *TA* and *TPC* concentrations.

We found significant differences ($\alpha$ = 0.5) in T%,R%,A% average (UV-Vis-IR regions) due treatments arising from the basic properties of the films as well as from the thickness differences. While, it is useful to compare the properties at the same thickness of the films, it would have been time consuming. Further, from the results reported here, we tend to consider that there would be an optimum thickness of a particular coatings to best serve to preserve a particular produce.

#### Optical reflectance (R%)

**Figure 2:**
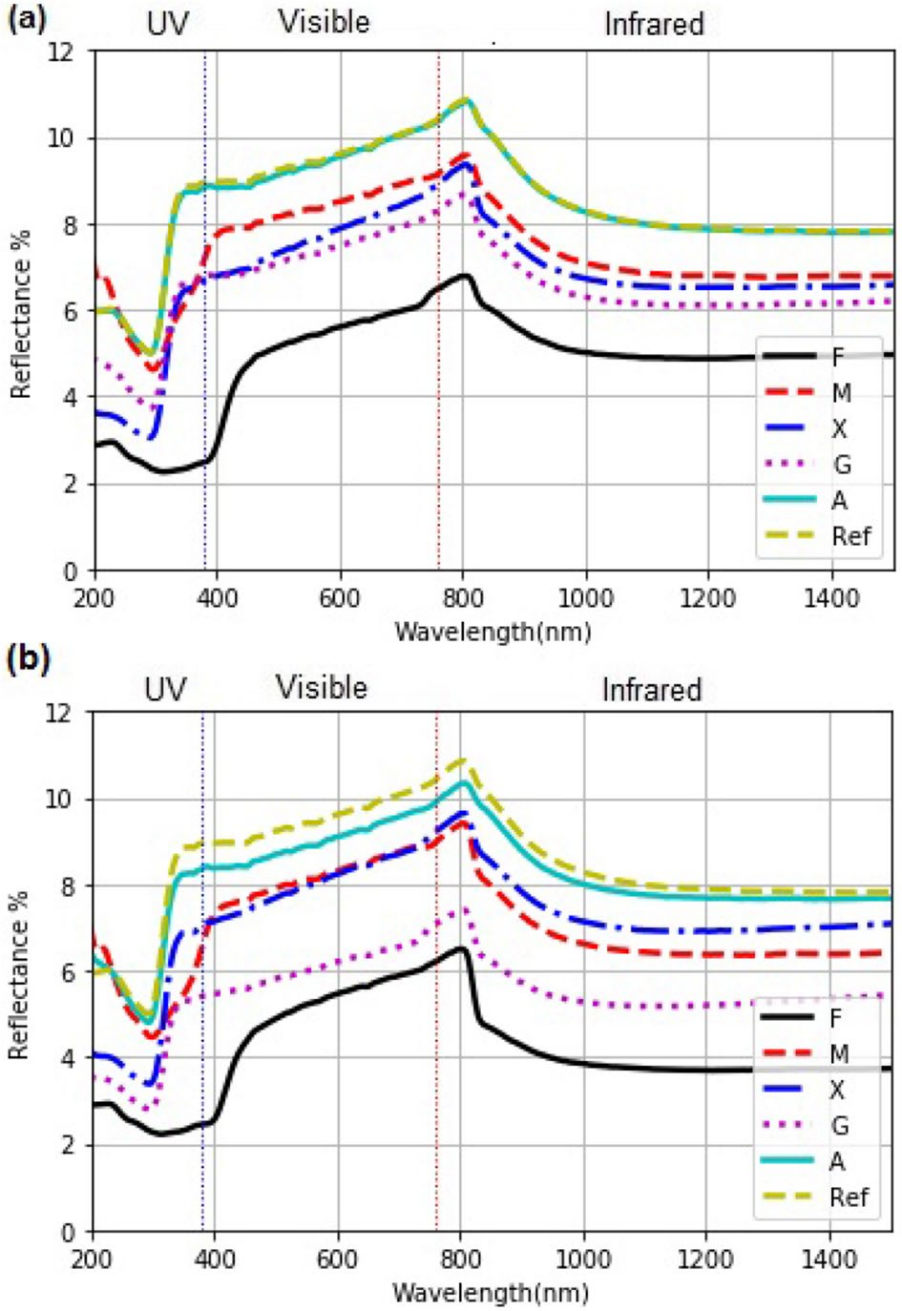
(**a**) Specular optical reflectance (R%) from the dried edible coatings cast on glass substrates from $4\upmu$l solution, and (**b**) from $8\upmu$L solution. The abrupt increase of R near 810 nm occurs because of a detector change in the spectrophotometer set at this wavelength. The rough surface of the coatings described in Fig. 3 introduces scattering, not captured equally by the detectors.

The reflectance ($R\%$) of each coating is observed in Fig. 2a and b, recorded using a glass slide as reference. The values of $R\%$ for the fenugreek coating (F), which has the highest thickness, is the lowest and for the guar gum coating (G), it is relatively higher (less scattering) for both 4 and 8 $\upmu$L. Overall, R% diminishes for 8 $\upmu$L coatings of M, F, A, and G. Thus, significant solar energy reflection loss exists in the 190 to 2500 nm wavelength interval with these coatings. Exception is observed for X.

For all the coatings, the reflectance falls for wavelengths below 450 nm. In the UV region in the spectral interval from 287 to 400 nm, the average $R\%$ is 6.0 for M, 2.5 for F, 4.5 for X, 6.8 for A, and 5.2 for G obtained in films using 4 $\upmu$L hydrocolloids. In coatings with 8 $\upmu$L, the $R\%$ is 5.6, 2.5, 4.9, 6.6, and 4.0 for M, F, X, A, and G, respectively.

**Figure 3:**
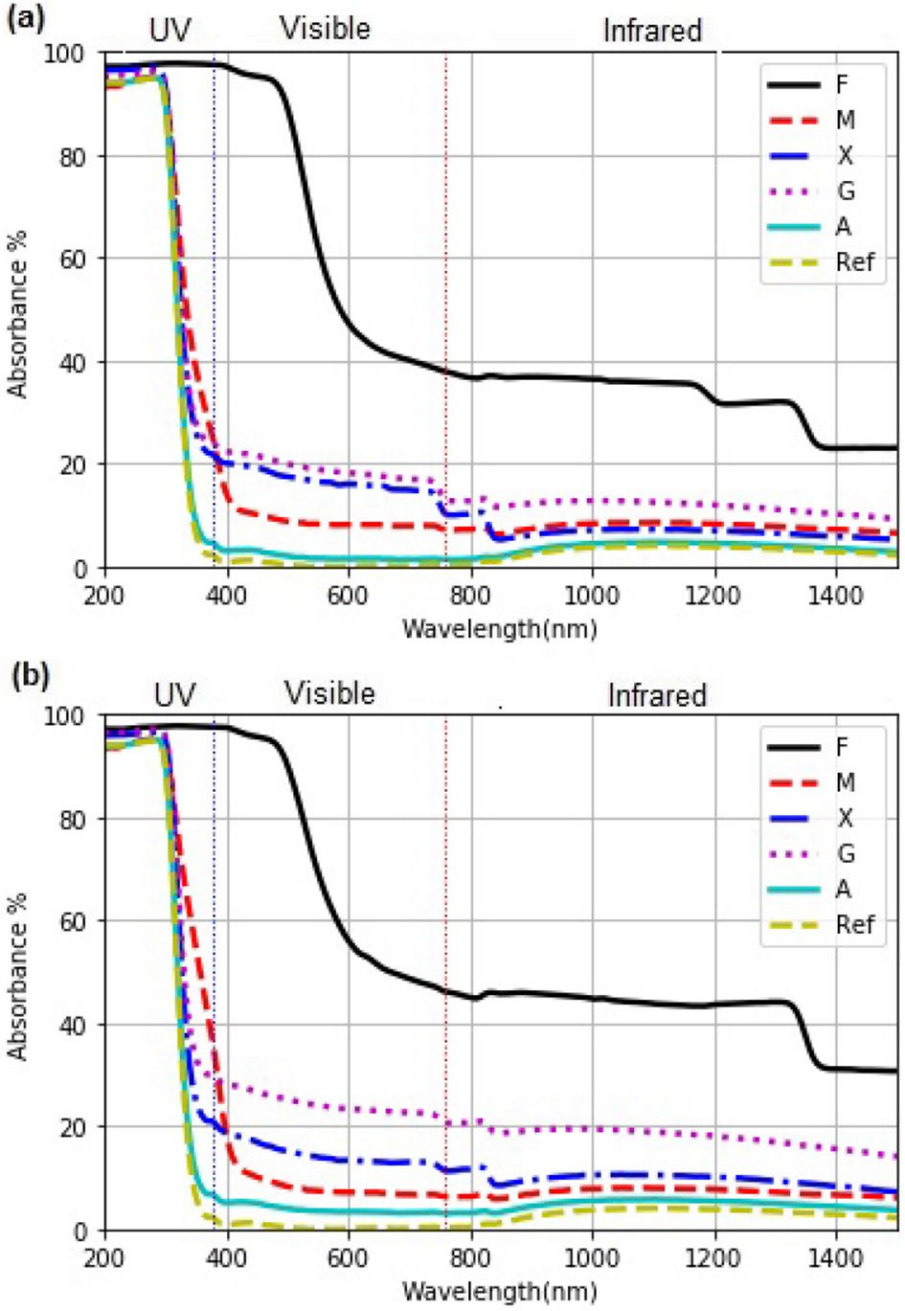
Percentage absorbance in the drop-cast coatings on glass slides made (**a**) 4 $\upmu$L and (**b**) 8 $\upmu$L of solutions.

#### Optical absorbance (A%)

In Fig. 3a and b is a comparison of A% of the hydrocolloid coatings obtained with samples of 4 and 8 $\upmu$L . For a higher volume used in the drop-casted coating, higher A% is observed, throughout the spectral range. Thus, solar irradiance will be better-absorbed with the increased coating ’s thickness leading to higher heating in the coating.

The gum Arabic (A), practically does not absorb light in the UV-A region (315 to 400 nm); the percentages are 4 and 7.5 for 4 $\upmu$L and 8 $\upmu$L, respectively. Thus, this coating does not prevent the interaction between the food sample and the UV-light in solar drying. Thereby, reactions promoted by UV-light during drying may occur.

On the other hand, M, X, and G show a partial UV-A light absorption. The average absorptions for M are 35% and 31%; for X, 19 and 22 %; for G, 21 and 29 % for 4 $\upmu$L, and 8 $\upmu$L, respectively. The partial absorption (and hence partial transmission) of solar UV-A may lead to enzymatic reactions through food-UV interaction, similar to enzymatic reactions which occur during ripening^46^. F-coating absorbs 100% of UV and visible radiation of wavelength $< 500$ nm (violet, and blue light). Thus, reactions promoted by this part of solar radiation may not take place inside the food product.

In the part of visible region, 600 nm to 780 nm, and infrared region (near and medium), a higher absorbance is observed for F and to lesser extent in A coating. Thus, we observe a reduction in the absorbance at longer wavelength, leading to a reduction in the gain of radiant energy gain in it. This has its effect in drying kinetic.

#### Optical transmittance (T%)

**Figure 4:**
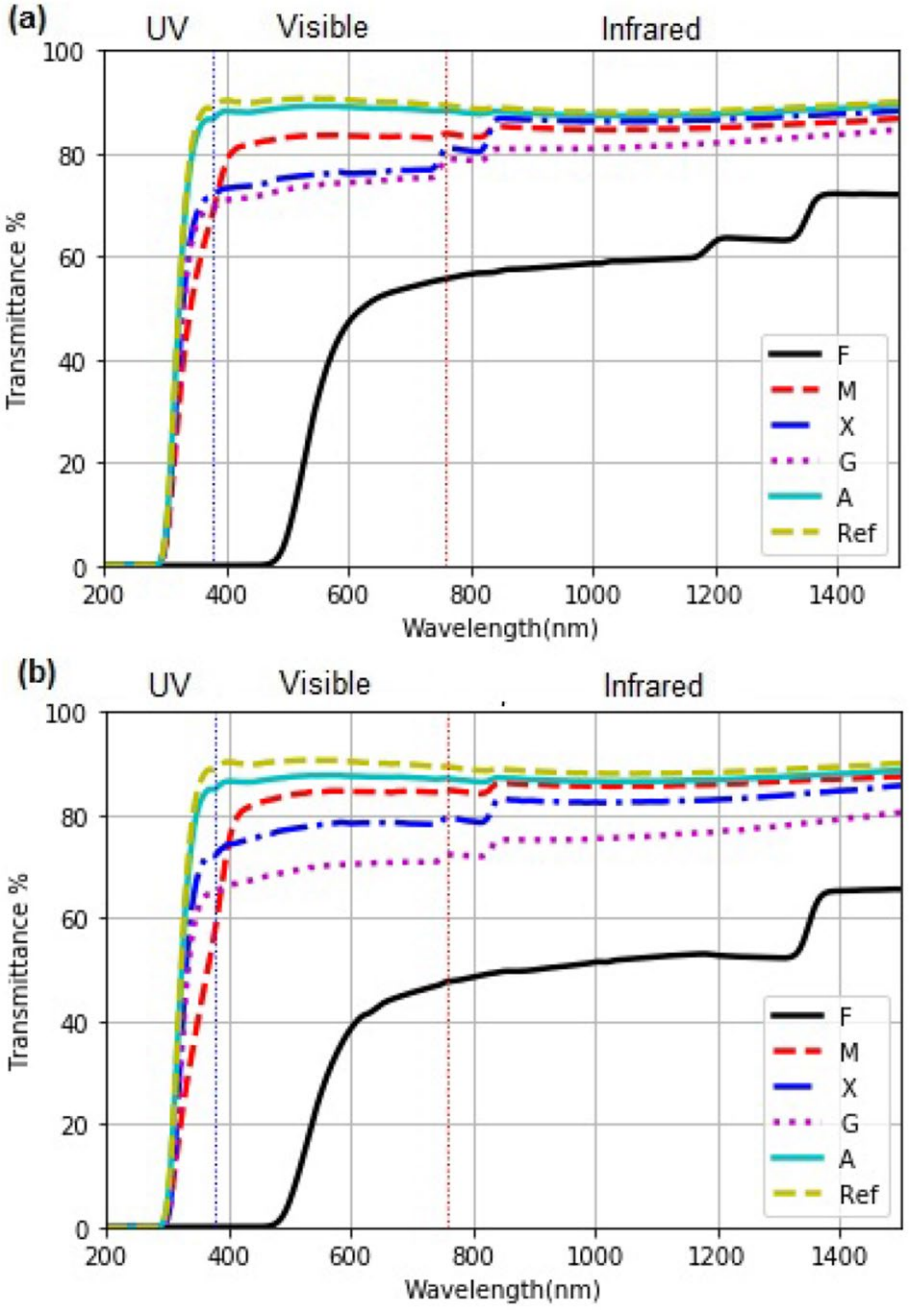
Optical transmittance of the coatings on glass substrates, dry-cast from (**a**) 4 $\upmu$L and (**b**) 8 $\upmu$L of the solutions.

In Fig. 4a and b, we present the optical transmittance for each coating. Generally these bear an inverse relationship with the volume of the hydrocolloids 4 and 8 $\upmu$L used in the drop-casting. The transmittance is highest with A and least with F.

In the UV-A region, a near-zero transmittance for F coating, and hence, the solar radiation will not contribute toward the enzymatic reactions promoted in this wavelength range. Nevertheless, the heat transference may affect the drying kinetic. For M, G, X coatings, the average transmittance in the UV-A region is 64 & 55%, 69 & 65%, and 75 & 72% respectively, for drop-casted coatings made from 4 and 8 $\upmu$L of the solution. Here, we can expect a role of solar UV radiation in enzymatic reactions.

In the visible range there is an increase in the percentage of transmittance for the coatings using 4 and 8$\upmu$L: M (81 & 83%), X (73 & 74%), A (72 & 68%) , G (70 & 78%). Again, we observe the lowest transmittance for F coatings. In the infrared region, the transmittance of M, G, X, and A ranges between 70 - 85%. Thus, energy from the infrared spectrum will influence the heating of the food sample. In the case of F, the transmittance in the near-infrared is below 60%.

### Thermographic experiments

**Figure 5:**
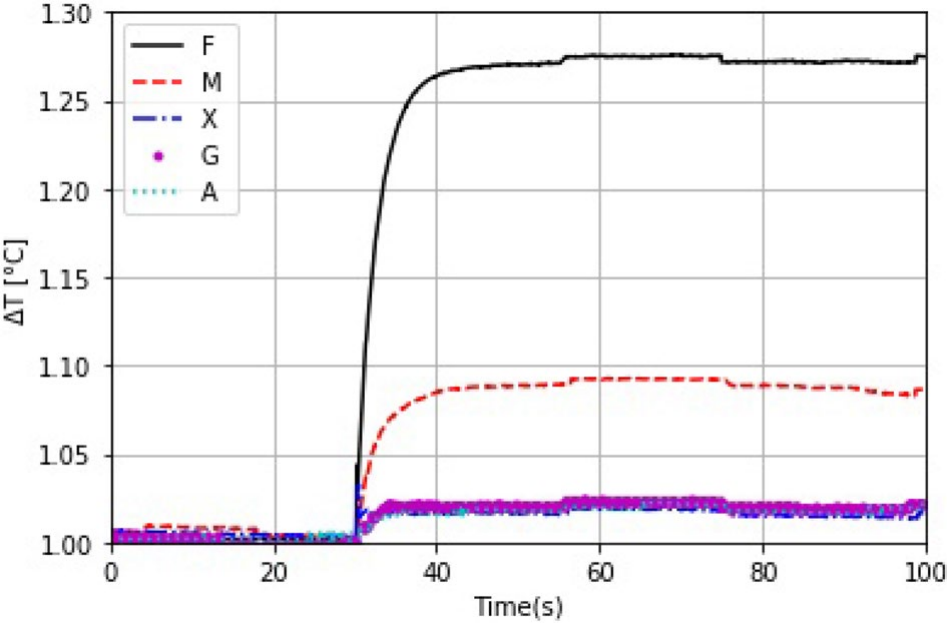
The normalized temperature change $\Delta T (^{\circ }C)$ recorded for the samples during 100 s period.

**Figure 6:**
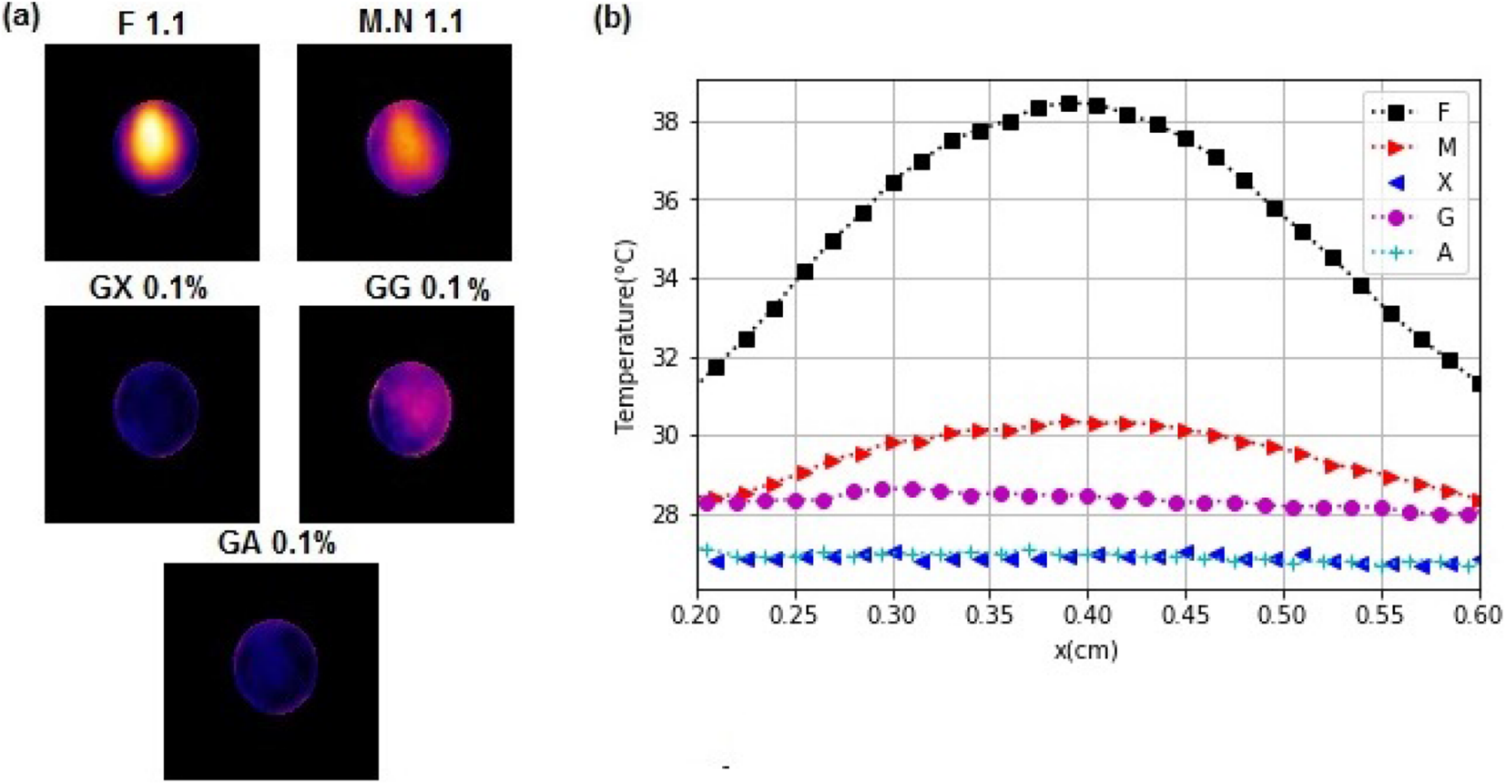
Thermal distribution. (**a**) Thermographic image for every sample and (**b**) temperature profile.

In the results on the optical absorbance Fig. (3) and transmittance (Fig. 4) of the coatings, we observed that most of the samples (except F) showed only minimal optical absorption, but have a high transmittance in the broadband from visible to infrared. When the samples are illuminated they absorb the light and after that they emit infrared radiation caused by this light mater interaction.This experiment allowed us to observe two aspects: first, the heating as a function of time; and the second, the heat distribution over the films. Figure 5 shows the change in the temperature of every sample. To compare thermal characteristics of the samples, we performed a normalization of the data using the measurement obtained under the same conditions for the coverslip. The graphical data shows that the sample F heats up 1.27 times faster than the glass slide, agreeing with the absorption curves. We observed the heat distribution over the samples, shown in Figure 6a. The optical absorption of the samples directly affects the temperature, and a rapid increase in temperature would have a direct influence on the drying kinetics (Fig. 6b). Thus, F and M coatings would lead to faster drying and the lowest may be for A (Arabic gum coating).

### Drying kinetics

**Figure 7:**
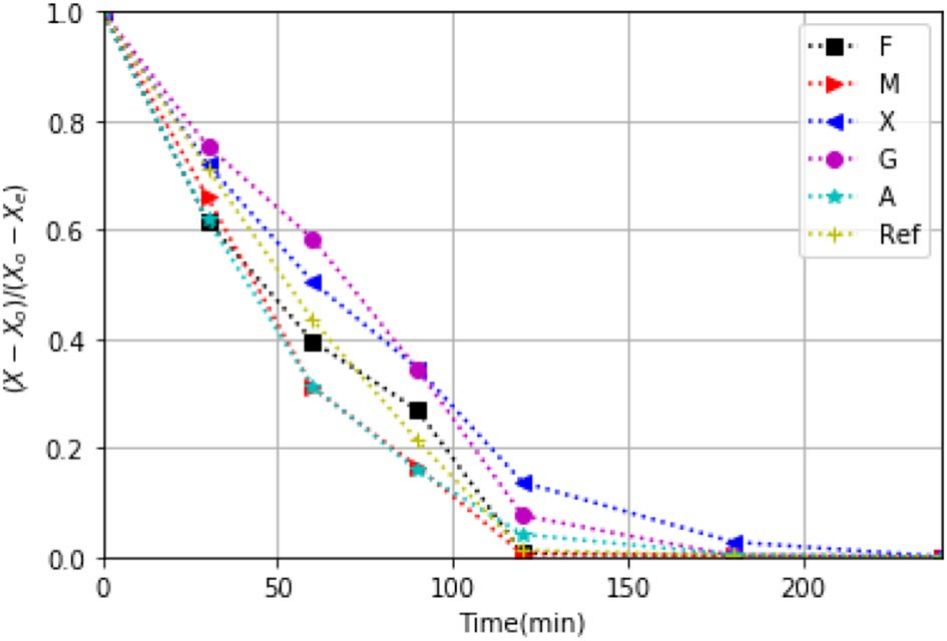
Drying kinetics for coated and uncoated (Ref) strawberry slices (2 mm in thickness) recorded for drying in an oven set at $60^\circ$C.

**Figure 8:**
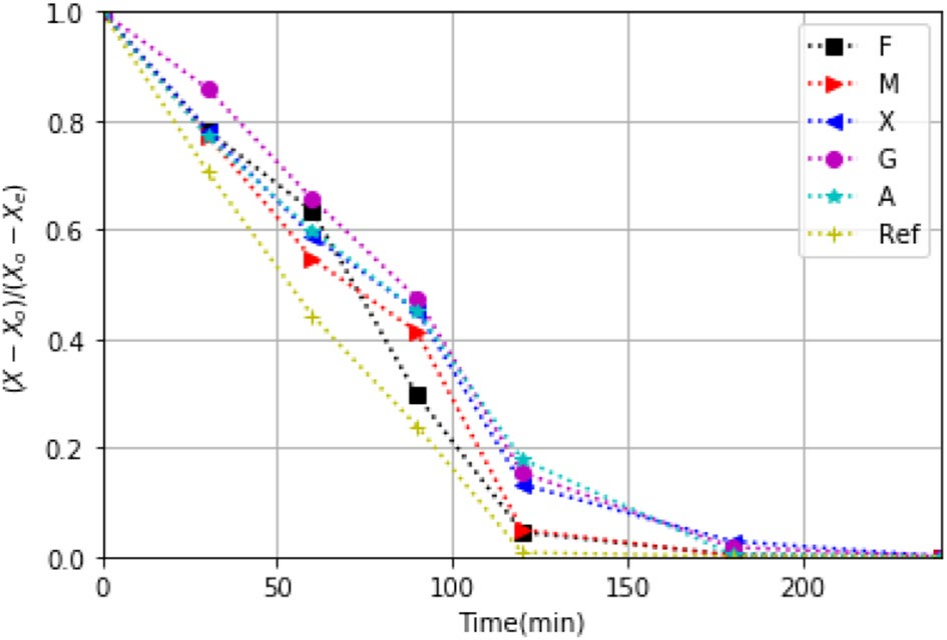
Drying kinetics under the solar drying process for strawberry slices (2 mm) with the edible coatings coating and without it (Ref).

The drying kinetics of strawberry with biopolymer coatings (M, F, X, A, G) and without such coatings (Reference sample: Ref) are shown in Figs. 7 and 8 for the oven-drying and the solar drying experiments. We found significant differences ($\alpha$ = 0.5) in drying kinetics due treatments. During the first 60 min of drying, a fast loss of mass occurs; water transfer from the coated strawberry to the surroundings takes place readily. During first part of oven drying, the moisture migration is slower in samples coated with X and G, and the quicker are with A and M. The behaviors of drying kinetics are influenced by the resulting temperature reached by the biopolymer coating according to Figs. 5 and 6b. Thereby, the major resistance to moisture transfer is obtained by X and G (Fig. 7). This is consistent with the drying velocity shown in the supplementary file (Fig. S3).

In the solar drying, the moisture transfer of X, G and A is slower than F, M and Ref. Besides, the high transmittance for X, G and A, suggests that the solar energy available for solar heating is not enough to obtain a faster dying. F is the coating that absorb UV, Vis, and infrared energy the most, holding the sample warm and thus promoting moisture transfer. Furthermore, the solar energy transmitted to the sample contributes to the heating of sample. This can be due to reflected energy shown in Fig. 2. The high heating rate and temperature reached (Fig. 5) with this coatings confirms the influence of the thermal characteristics in the mass transfer during drying. Also, the higher thickness, and the isotropic roughness with F and M, lead to good thermal conductivity via both coatings, and this favors the mass transfer during the drying. The low optical reflectance obtained with F, also leads to a higher heating rate for strawberry slices with this coating. Therefore, if moisture transference is the decisive criteria in choosing hydrocolloid coating for solar drying, F seems the edible coating of choice. However, nutritional value of the product also is of importance, and hence the optimum choice has to wait.

The differences between the drying kinetic of moisture can be explained trough the properties of the films, such as permeability, porosity, film structure, and the vapor transport from within the sample to the edible film and through it to surroundings. At the beginning of the drying, the dominate mechanism is vapor transfer by convection. At this stage, all films have a similar behavior. Such behavior repeats at the end of the drying process as well. Thus, at the beginning and at the final stage, the hydrocolloids properties do not influence the moisture-drying kinetic. These properties influence the kinetic in the intermediate drying stage. Also, this behavior is according to the drying rate (Fig. S3). This distinction arises from the properties that are influenced by the moisture content; the elongation capacity, tensile strength, tensile elongation, optical transmittance, and water vapor permeability; also, the coating can be consider as insulating, creating temperature barriers^47^. Moisture contributes to plasticizing effect for the coating, because water is in contact with the polysaccharides, influencing the chain mobility.

The moisture content (X$_{dm}$) at the end of the solar drying (360 min all) of each test is: 0.14, 0.13, 0.13, 0.13, 0.15, 0.15 kg$_w$/kg$_{dm}$ (kg of water per kg dry mass) for Ref, M, F, G, A, X, respectively.

### Anthocyanins

**Figure 9:**
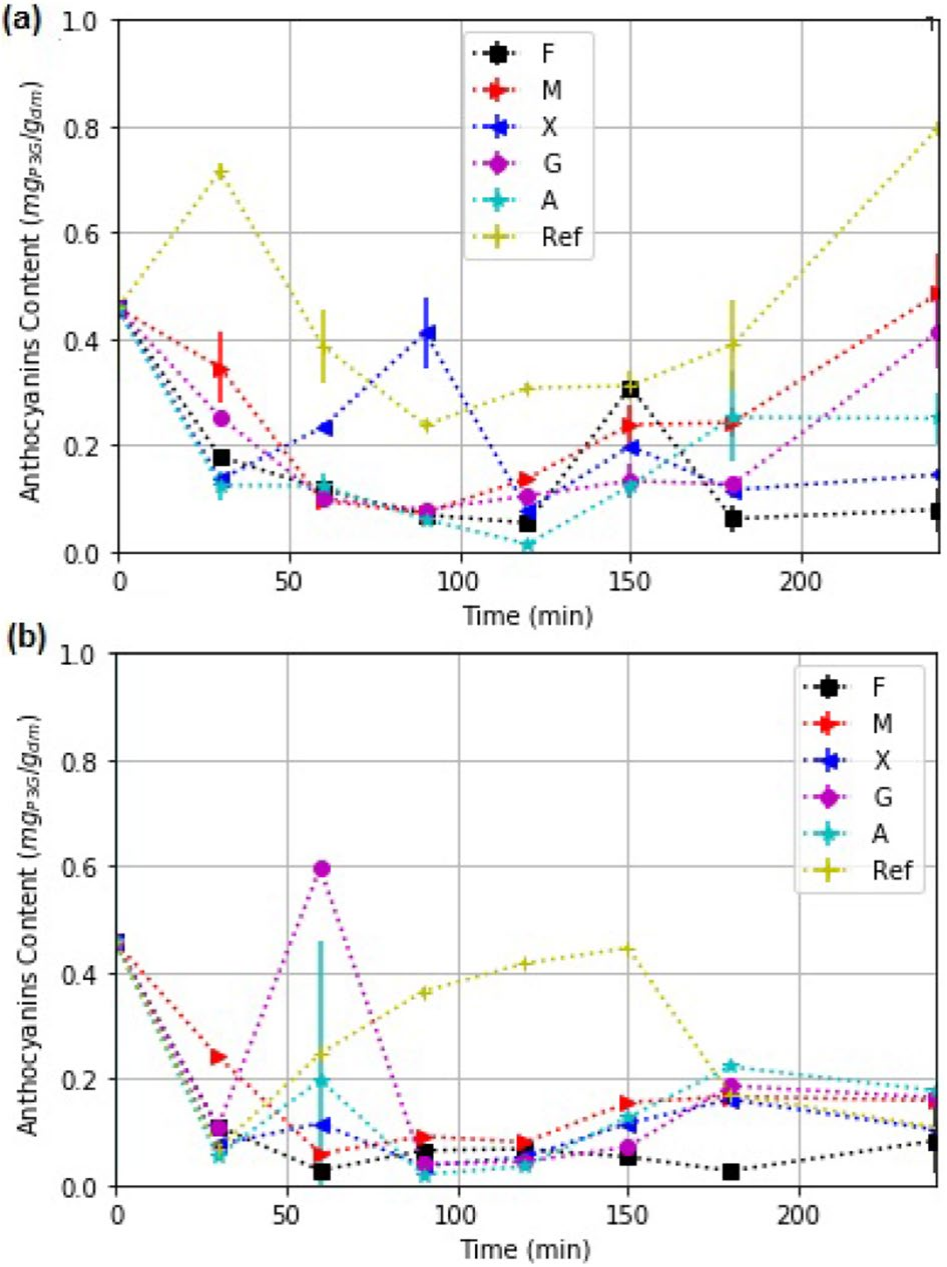
Kinetics of total anthocyanin content for strawberry slices with edible coatings (A–F) and for the reference slices without such coating under: (**a**) solar drying and (**b**) oven-drying.

Anthocyanin kinetics of coated strawberry slices used in the solar and oven drying are shown in Fig. 9. Each drying test was carryout during 360 mins.We found significant differences ($\alpha$ = 0.5) in total anthocyanin due treatments and drying time. In solar experiments, there is an increase in the total anthocyanin content (TA) along the drying process. This increase may be related to the temperature of processing^48^, but the relative increase with respect to the oven-dried slices suggests that the presence of the UV component of the solar radiation plays a role. The concentration of anthocyanins in the food product may be affected by oxygen, pH, metallic ions activity^9^ temperature, light, UV radiation^8,9^. During solar drying, the sunlight (UV-light, Vis-light, infrared-light), temperature and oxygen can also act over this concentration. Different studies indicate a degradation of anthocyanins due to UV-light, incandescent light and IR-light^49^ and UV-C light^50^. Recent results indicate suggest that the final concentration of total anthocyanin (TA) in solar dried blackberry pulp is better preserved without solar irradiation. However, when the samples were solar-dried under UV, a higher TA content was obtained with high percentage of the UV irradiation^51^. In our study, time dependency of TA was studied, the UV irradiation favored the TA concentration in solar dried samples.

For the slices with fenugreek coating (F), the UV component has been totally filtered off, and the TA is the least. The highest TA is for the strawberry slices dried without any coating in the solar drying. This illustrates the role of solar irradiation to maintain the TA content in the dried slices (0.14 kg$_w$/kg$_{dm}$) at the same level as in the fresh produce for only certain edible films.

There are many reports on the likelihood of enzymatic reactions promoted by the processing temperature, leading a degradation/formation of anthocyanins^4,52,53^. We note that the time dependent TA-content in the produce being dried results from a balance between the syntheses and degradation of anthocyanins in it^54^. In solar drying, formation of anthocyanins may be promoted by partial absorption of UV irradiation, similar to that reported for UV-A and UV-B absorption in the deep cell layers^9^.

We note an increase in TA for strawberry slices with M and G coatings at the final stage of drying. The UV received in the slices is between 64 to 55% for M and 69 to 65% for G of the total UV irradiance. The differences between Ref and G and M are due to the solar irradiance received by the sample. The effect of UV-irradiation on secondary metabolites and enzymatic activity in the product derives into two main opinions (a) the UV light in processing may influence the concentration and/or enzymatic activity^55^, and (b) the UV light in processing does not influence the concentration and/or enzymatic activity^56^. In Beaulieu^56^ experiments, a UV-source of intensity 3000 $\upmu$W/$cm^2$ at 313 nm was used. Lante^55^ observed, a decrease in PPO enzymatic activity with the UV-light exposure, contributing to a decrease in darkening. In our experiments, the entire solar spectrum influences the experiments, and a partial blocking though edible solar filter was studied.

We assign the reduction in TA observed in strawberry slices with fenugreek coating (F) to the absence of UV irradiation in the drying slices due to the optical absorption in the coating (100 % for UV-Blue light). The absence of UV reduces the synthesis of TA in the slices. Also, we observe that the partial absorption of the green-red light (60%), and near-infrared (43%) promote the heating of the coating and the sample and therefore, damages the anthocyanin structure, as proposed by López-Ortiz^4^, thereby reducing the TA concentration. Thus, the total blockage of the UV content in solar drying of coated strawberry slices is not desirable. The partial increment in TA during the last stage of drying may be due to the heating of the sample. The enzymatic activity was not evaluated in the present work. In the experiments with X and A, a low concentration of TA resulted.

In convection drying, the final concentration of TA is less than the initial TA concentration for all samples. This can be due to the relative overheating of the samples ($60^\circ$C). TA increment was found in Ref and G at intermediate drying stage. The partial increase can be due to temperature effect as a reported in heating processing^57,58^. At the end of drying, TA decreased in all samples.

### Total phenolic compounds

**Figure 10:**
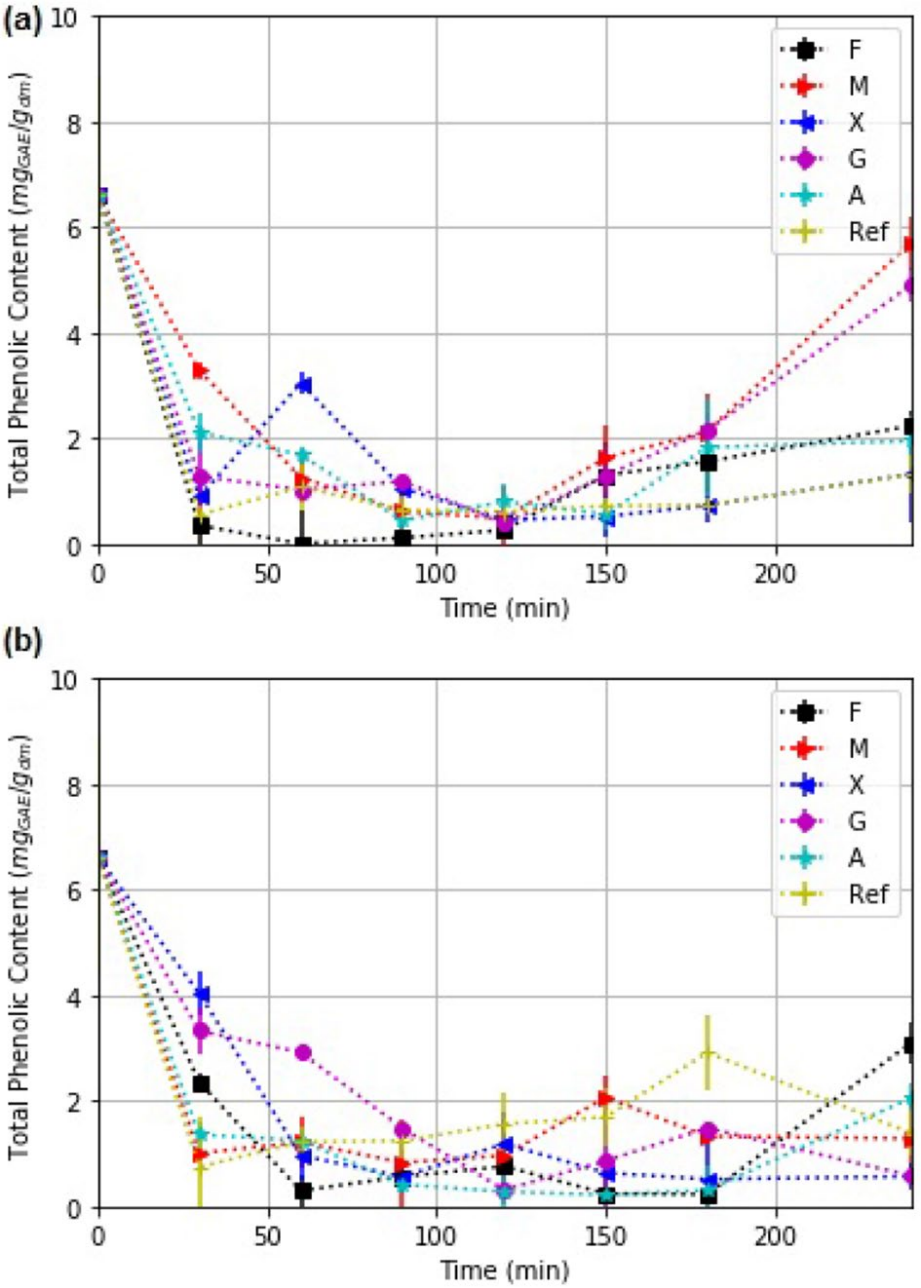
Kinetics of total phenolic compounds in strawberry slices with edible coatings and without it (Ref), studied for (**a**) solar drying, and (**b**) oven-drying.

Total phenolic compounds kinetics of strawberry slices with and without coating is shown in Fig. 10. We found significant differences ($\alpha$ = 0.5) in total phenolic compounds due treatments and drying time. The concentration of total phenolic compounds (TPC) at the end of drying is less than the initial concentration in all cases for solar and oven drying. Thermal degradation of phenolic compounds has been previously reported^59–62^. Nevertheless, in solar drying, we observe an increase in TPC concentration at last stage of drying, similar to the behavior obtained in TA. The differences between the results are attributed to the solar irradiance received by the food sample.

In solar drying, low TPC values were obtained with Ref, X, F and A coating, which is consistent with the results on TA (except for Ref). The differences in the behavior between TPC and TA arises in that the increment in TA was not enough to influence the TPC accumulation. Similar to the results on TA, M and G registered an increase in TPC at the last stage of solar drying.

The present results are in agreement with the differences between the percentage of UV-light received by the food sample and that reported in the literature on UV effect^7,63^. Nevertheless, there are other factors such as molecular size, conformation and inter-and intra-molecular interactions of polysaccharides that influence the functionality of the coatings and should be studied further.

### Relevance of the work

Strawberries are an important source of nutrients, minerals, vitamins and bioactive compounds stated in %: proteins 0.67, lipids 0.30, carbohydrates 7.68, fibers 2.0, sugars 4.89, calcium 0.16, magnesium 0.13, phosphorus 0.24, potassium 1.53, Vitamin C 0.058, folate 0.024, choline 5.7, vitamin A 0.001, Lutein + zeaxanthin 0.026, Vitamin K, phylloquinone 0.02 among others^64^. Their consumption promotes human health and beneficial effects due the bioactive compound content, and among this are anthocyanins and polyphenols. The ingestion of anthocyanins is reported to help lower blood pressure, improve visual acuity, reduce cancer cell proliferation, inhibit tumor formation, prevent diabetes, lower the risk of CVD modulate cognitive and motor function as well as promote anti-inflammatory and anti-bacterial activity^65^. However, their low stability during processing and storage calls for particular attention^66^ during solar drying. Likewise, hypothesized/evaluated protective effects of polyphenols in acute and chronic diseases, including obesity, neurodegenerative diseases, type 2 diabetes, and cardiovascular diseases and the need to assure stability of these nutrients during processing of the produce is reported^67^. In this report we find that the drying process of strawberry slices for storage and subsequent ingestion does involve changes in the bio-compounds content. We would continue the investigation focusing on the best coating identified here.

## Conclusions

We observe differences between optical properties of edible coatings prepared from hydrocolloid solutions and applied on strawberry slices for solar drying and oven-drying of the food product. Coatings with fenugreek solutions (F) showed a high UV absorbance and practically zero transmittance. These characteristics give it excellent functionality to be used as coating with solar filter properties. The optical properties are influenced by coating thickness. In general, the anthocyanin content and total phenolic compounds content are better preserved in solar experiments than in the stove-drying due the solar filters. A partial transmittance of UV light is recommended to obtain an increment in TA and total phenolic compound (TPC) contents. The total absence of UV is not recommended for drying in which TA and TPC are involved, which makes F-coating less attractive from nutritional (antioxidant) point of view. In the case of M, an optimum case is noticed, the slices dried at 120 min, and preserved TA and TPC in a satisfactory manner. Both F and M may have anti-glycemic activity, known in traditional medicine, but requires further evaluation.

## Methods

### Sample preparation

We buy commercial strawberries (*Fragraria vesca*), cladodes of Opuntia (*Opuntia ficus-indica*—called nopal in Mexico, and called so in this report) and fenugreek seeds (*Trigonella foenum-graecum*). This study was carried out with the relevant institutional, national, and international guidelines and legislation . The nopal was selected according to NMX-FF-068-1988, size (Class D 13.0–16.9 cm), and color (without brown pigments), without defects (mechanics, physiologic, noxious wildlife). Fenugreek without discoloration was selected, without extraneous matter. After removing the spines from the cladodes, these were washed in water to eliminate impurities. Also, we washed and rinsed the fenugreek. We washed and disinfected the strawberries with a 1% sodium hypochlorite solution. These were sliced in cross-sectional cuts with a stainless slicer to two mm in thickness. The slices were coated by immersion for 3 s in hydrocolloids of the biopolymers. We tested five biopolymers: nopal mucilage (M), xanthan (X: Fig. S1a), gum Arabic (A), guar gum (G: Fig. S1b) and fenugreek mucilage (F: Fig. S2). We keep a control sample set of untreated strawberries for comparison. All experiments were done in a triplicate set of samples for each case.

### Preparation of the coating solutions

Past work on edible coating on food often reported the quantity of each component used as a molar ratio^68^, or in volume-by-volume % v/v^68,69^, or as weight-by-volume % w/v^68–73^. Based on these, we prepared the edible coating solutions for this work with v/v and w/v relationship.

We obtained the Opuntia and fenugreek mucilages through maceration (1:1) at $80^\circ$C, as described previously in López-Ortiz^74^. We prepared individual aqueous solutions to 0.001 g/mL of xanthan gum, gum Arabic, and guar gum, using an analytical balance (Scout Pro, Ohaus, 108USA). The xanthan gum and gum Arabic were slowly added to distilled water at $80^\circ$C, and guar gum was added to distilled water at $90^\circ$C. The solutions were mixed well for 20 mins in a vortex shaker.

For the application of edible coating the most used methods are: dip-coating^69,73^ and brush painting with filbert-shaped bristles^72^. In our studies, we use dip-coating. The effect of thickness variation on the edible coating’s optical and thermal properties and their effect on total anthocyanins and total phenolic compounds would have improved trough optimization of the coating. However, that implied much more samples for each coating material, and hence multiple sample sets. In this present work, we want to obtain the tendency of the coatings’ behavior concerning optical and thermal properties. For each type of hydrocolloid coating, there will be an optimum thickness to assure the intended shelf-life without adversely affecting/modifying the nutritional value of dried food products. Thus, we hope that future work will arrive at such definite results for each coating - thickness versus shelf life and quality retention (color, total anthocyanins, total phenolic compounds, etc.).

### Optical properties

We determine the optical properties of dried coating solutions cast on Corning microscope glass slides ($25 \times 76 \times 1$ mm) using a spectrophotometer (UV-3600C, Shimadzu, Japan). We measured the optical properties of edible coatings dop-cast on Corning microscope glass slides and dried. This permitted the films to have a smoother surface leading to more reliable optical reflectance measurements in UV-Vis-IR spectral regions. We evaluate coatings of two different thicknesses prepared from 4 $\upmu$L and 8 $\upmu$L of the coating solutions drop cast on horizontally kept glass slides. First, the 4 $\upmu$L coatings were introduced into a laboratory oven, set at $60^\circ$C for two h. On some of the dried 4 $\upmu$L coatings another layer was drop cast and the drying was repeated. These are 8 $\upmu$L coatings. We measure optical transmittance, absorbance, and reflectance for all samples, 4 and 8 $\upmu$L samples on the glass slides (Fig. S4).

The coating thickness was usually measured in past reports by randomly positioning a vernier caliper or digital micrometer^70,75^. In our case, we measured the thickness of all the edible coatings on the glass slide by utilizing the glass slide-to-coating step using an Ambios XP 200 Profilometer. We performed the measurement with 5 mg-force, scanned over a length of 2 mm at a scan rate of 0.02 mm/s.

### Thermal behavior

**Figure 11:**
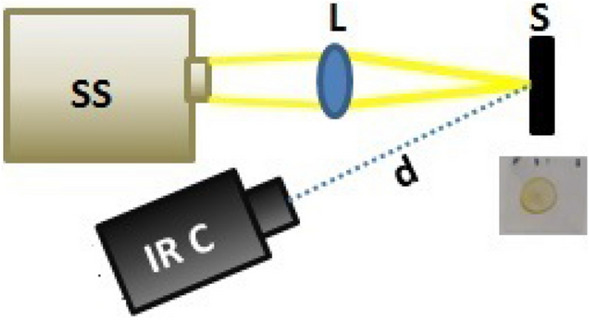
Set up of the thermographic experiments.

To carry out the thermographic experiments, we placed the strawberry slices with the coatings on microscope cover glass slides (coverslips) of 125 $\upmu$m in thickness. Figure 11 shows the experimental set-up. The thermographic camera employed for the experiments is a FLIR $\times 6540$sc with a spatial resolution of $640 \times 512$ pixels, which uses an indium antimonide (InSb) detector. We placed it at a distance of 42 cm from the samples. The camera detection range is 1.5-5.5 $\upmu$m mid-wave infrared band, such that it can detect temperature differences of 20 mK. For these experiments, we used a lens, MWIR 50 mm 1:2.0 USL, in the camera. To illuminate the samples, we used a white-light lamp of 200 W (Newport Hg-Xe) directed toward the free-standing sample’s frontal side to induce a thermal wave. The radiant emission from our illumination source goes from the Ultraviolet to the near-infrared. The illumination of the samples with the lamp would not be enough for heating the samples, or else, a longer exposure time would be required. Thus, we found it necessary to focus the light from the lamp on to the sample using a lens of focal length, $f = 20$ cm. The thermographic images were recorded at 30 Hz, by detecting the temperature increase in the sample during a 70 s period. We performed the measurements under ambient conditions without using a vacuum system. However, we covered the samples by placing them in a thermal isolation chamber to avoid modifications by air- currents. This chamber avoids external radiation, which may otherwise hit the thermographic camera, and distort the pattern recorded. It also maintains the whole system at a constant temperature throughout the measurement by avoiding air currents.

### Drying description

**Figure 12:**
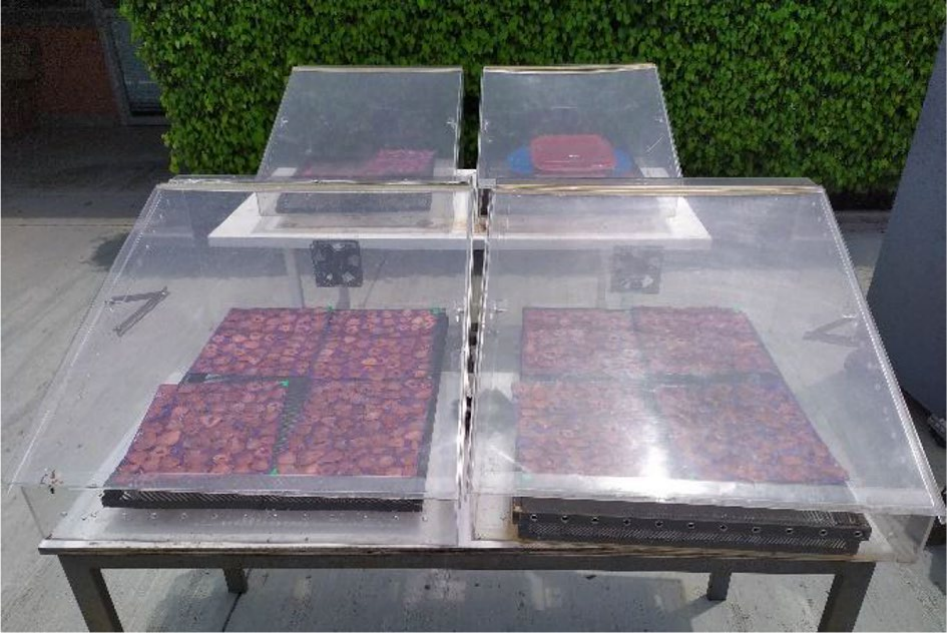
Cabinet-type direct dryer system used in experiments with coated and uncoated strawberry slices.

We use a cabinet-type direct solar dryer system^76^ for all experiments (Fig. 12). The air temperature inside the dryer was $50^\circ$C on average during 10:30 to 4:30 pm local time (Fig. S5). The maximum temperature recorded inside was $60.1^\circ$C. The temperature inside drying chamber was measured with a thermometer (37,950, Cole-Parmer, USA). We use a passive mode in the experiments - without forced convection. The solar irradiance, relative humidity, and ambient temperature at our site was recorded by the meteorological station (ESOLMET, IER-UNAM, Temixco) through a data acquisition system (CR1000, Campbell Scientific, USA). We placed the coated slices on plastic meshes (27.2 x 34.4 cm) and introduced them into the driers (Fig. 12). The plastic mesh with the slices was weighed at intervals for studying the drying kinetics. The control drying experiment was done at $60^\circ$C in an oven tray drier (DKN402C, Yamato-Japan). In order to avoid differences caused by weather variations, the solar drying experiment was done on all the samples simultaneously.

### Drying kinetics

We use the stove method 972 (AOAC, 2012) for the determination of the moisture content of the product after elapsing a time (t) from the weight loss upon drying, using an analytical balance (Scout Pro, Ohaus, USA) with a precision of 0.01 mg. The final recorded weight was used as the equilibrium moisture content ($X_e$). We calculate the normalized moisture content (MR) as follow:

1 $$\begin{aligned} MR = \frac{(X-X_e)}{(X_0-X_e)}, \end{aligned}$$

where *X* is the moisture content of the sample at any drying time, $X_0$ is the initial moisture content.

### Chemical properties

In this section we describe the methods used to extract the organic compounds, determination of anthocyanins, and phenolic compound.

#### Organic extracts

We use a methanol–water (8:2) solution for total anthocyanins (TA) extraction, and methanol–water (8:2) acidified (0.1%) for total phenolic compounds (TPC) extraction. The extraction process was assisted by ultrasonic bath for 30 min. The total organic extract (TOE; Fig. S6c) was recovered using a cotton-packed Pasteur pipette. It was transferred and stored at $4^\circ$C in an amber colored vial prior to performing the TPC and TA analyses (Fig. S6).

#### Determination of total anthocyanins (TA)

Cyanidin 3-glucoside, Pelargonidin 3-glucoside, and pelargonidin 3-rutinoside are the main anthocyanins found in strawberries^77^. The method described by Lee et al.^78^ is used for measuring TA-content in a material. The molecular mass used in the present procedure is 449.2g/mol for Cyanidin 3-O-glucoside (C$_{21}$H$_{21}$O$_{11}$ $^+$). Therefore, we use the modified pH-differential method to determine the total monomeric anthocyanin content^78^. For this, potassium chloride (0.025 M, pH 1.0) and sodium acetate (0.04 M, pH 4.5) solutions were used as the buffer system. Optical absorbance measurements were made for samples at two pH values (1 and 4.5) for radiation of wavelengths, 510 nm (maximum intensity for visible light - vismax) and 700 nm, in each case. The absorbance differences (*A*) of anthocyanins follow from Eq. (2):

2 $$\begin{aligned} A = (A_{vismax} - A_{700nm})_{\text {pH}_{1}} - (A_{vismax}- A_{700nm})_{\text {pH}_{4.5}}. \end{aligned}$$

Equation (3) gives the concentration of anthocyanins ($C_{TA}$) in mg/L:

3 $$\begin{aligned} C_{TA} = \frac{A \times MW \times DF \times 1000}{1 \times \epsilon }, \end{aligned}$$

Here, *MW* is the molecular weight of 449.2 g/mol of anthocynins; $\epsilon$ is its molar extinction coefficient (26,900 $L/mol \cdot cm$); and *DF* is the dilution factor (= 1 in this work). To express the concentration of *TA* as cyanidin-3-glucoside equivalents per gram of dry matter (mg$_{C3G}$/g$_{dm}$), we use Eq. (4).

4 $$\begin{aligned} TA= \frac{C_{TA} \times V_{s}}{1000 \times m}, \end{aligned}$$

where $V_s$ is the final volume (ml) of the organic extract and m is the mass expressed in gram of dry matter.

#### Determination of total phenolic compounds (TPC)

We use the modified Folin–Ciocalteu colorimetric method according to Méndez–Lagunas^53^ for TPC determination, and use gallic acid equivalent (0-36 mg$_{GAE}$/100 mL) as standard. We measure the absorbance at wavelength 750 nm in a spectrophotometer (Genesys 10s Uv-vis, Thermo Scientific, China) to obtain $C_{TPC}$ in $mg_{GAE}/ 100$ ml; and we transform them to volume equivalent units by eqn. 5:

5 $$\begin{aligned} TPC = \frac{C_{TPC} \times V_{s}}{100 \times m}, \end{aligned}$$

where *TPC* is the total phenolic concentration (mg$_{GAE}/ g_{dm}$).

### Statistical analysis

We perform an analysis of the variance (ANOVA)^79^ considering the effect of the treatment (F, M, A, G, X, Ref) on answers variables (optical properties, thickness, drying kinetics). Additionally, we perform an ANOVA considering the effect of the treatments and drying time on TA and TPC. We tested the probability to observed differences among the experiments. When the calculated probability is less than of the chosen significance levels, we identify variations among the treatments. The absence of variations occurs when the calculated probability is more than the chosen significance levels, and thereby we assume that no significant differences exist among the treatments or the effect of treatments are statically equivalents. A value for $\alpha =0.05$ is used in all data analysis. The NCSS 2020 software Version 20.0.3. is a tool used for the comparison of the variances.

## Supplementary Information

Supplementary Information.
