# Supplementary material for: Optical and thermal properties of edible coatings for application in solar drying
Source: Sci Rep. 2021 May 12;11:10051. doi: 10.1038/s41598-021-88901-5 (PMC8115689; doi:10.1038/s41598-021-88901-5)
Supplement: Supplementary file 1 — Supplementary Information. [file 41598_2021_88901_MOESM1_ESM.pdf]

Supplementary: Optical and thermal properties of edible coatings for application in solar drying

April 5, 2021

López-Ortiz, A.[1] Pacheco Pineda, I.Y.[2] Méndez-Lagunas, L.L.[3] Balbuena Ortega, A.[1] Guerrero Martínez, Laura[1] Pérez-Orozco, J.P.[2] del Río, J.A.[1] Nair P. K.,[1]

[1]Universidad Nacional Autónoma de México, Instituto de Energías Renovables, Morelos 62580, México

[2]Instituto Tecnológico de Zacatepec, Departamento de Ingeniería Química y Bioquímica, Tecnológico Nacional de México, Morelos 62780, México

[3]Instituto Politécnico Nacional, Centro Interdisciplinario de Investigación para el Desarrollo Integral Regional (CIIDIR), Oaxaca 71230, México.

a) 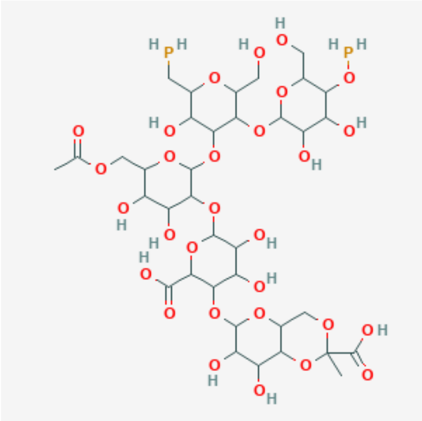

b) 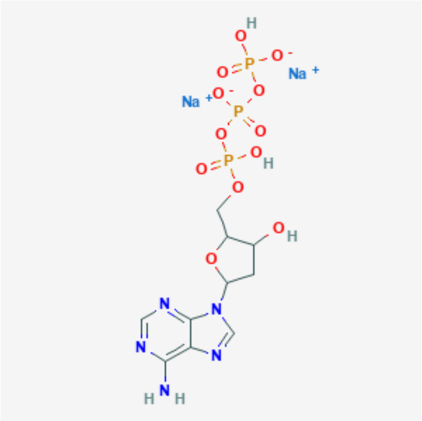

S 1: a) Xanthan gum structure Pubchem identifier: CID 131750926, https://pubchem.ncbi.nlm.nih.gov/compound/131750926#section=2D-Structure; b) Guar gum structure. Pubchem identifier: CID154724046, https://pubchem.ncbi.nlm.nih.gov/compound/154724046#section=2D-Structure

Xanthan gum consit in two mannose units, and one glucuronic acid unit, in the molar ratio 2.8:2.0:2.0, and molecular weight distribution between 2 and 20 million Da.[1]

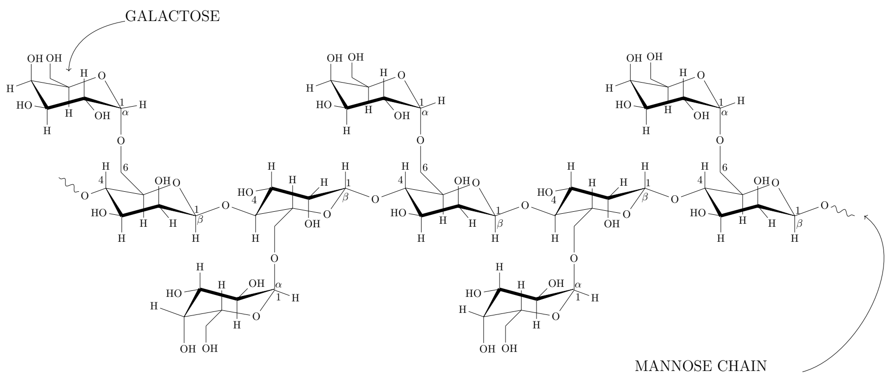

S 2: Fenugreek gum structure. (Own drawing, modified from [2])

Fenugreek gum is a galactomannans compound 2, that is composed by mannose and galactose groups. Galactomannan is a carbohydrate (soluble fiber) fraction representing 45–60% of the fenugreek seed. In fenugreek gum the relation is mannose:galactose (1:1) [2]. Fenugreek contain proteins, amino acids, flavonoids, steroidal saponins, coumarin, lipids, vitamins, minerals, galactomannan fiber, and alkaloids, such as trigonelline [3]

Gum Arabic (G-A) is mainly composed by two polysaccharide components and a glycoprotein, with 90% carbohydrate. The carbohydrate fraction of G-A is galactose, rhamnose, glucuronic acid, and Arabinose. Also, G-A contain essential and trace elements like aluminum, phosphorus, magnesium, copper, zinc, and iron [4]

Opuntia mucilage is a polysaccharide composed by 21–40.1% of galactose; 7–13.1% of rhamnose; 8–12.7% of galacturonic acid; 24.6–42% of arabinose; and 22–22.2% of xylose [5]

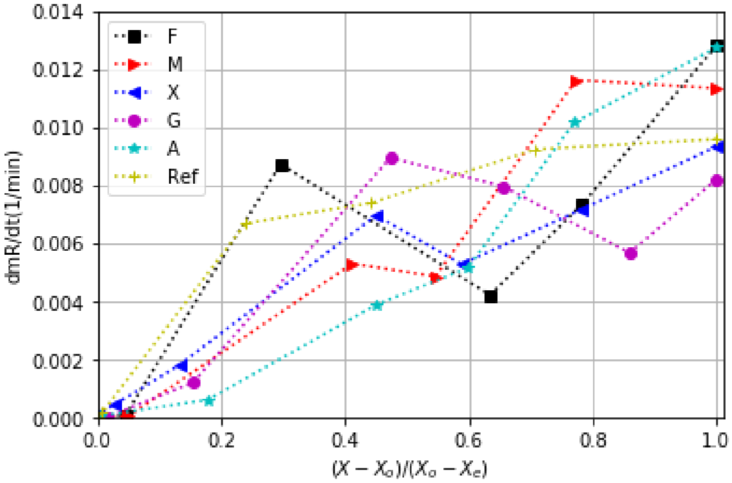

a)

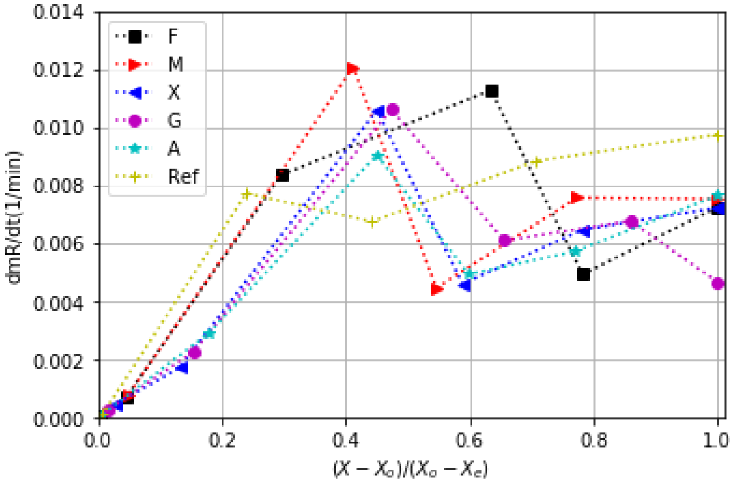

b)

S 3: Drying rate a) stove drying process; b) solar drying process

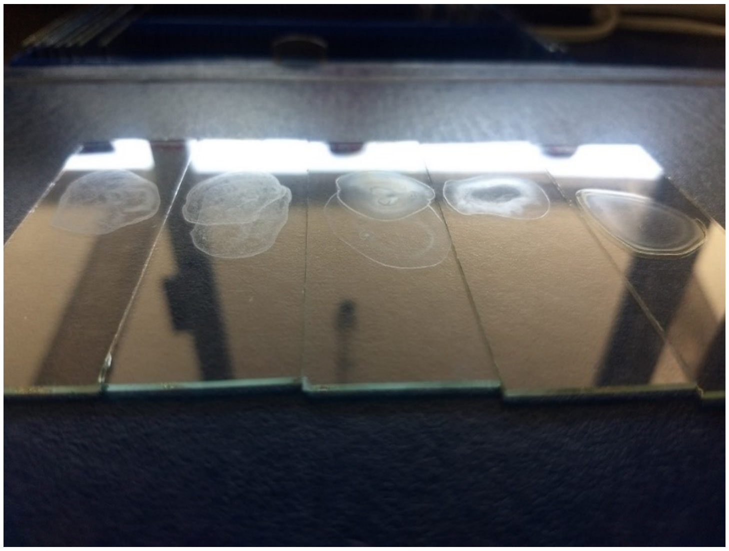

S 4: Coating solutions laid on glass for optical measurements

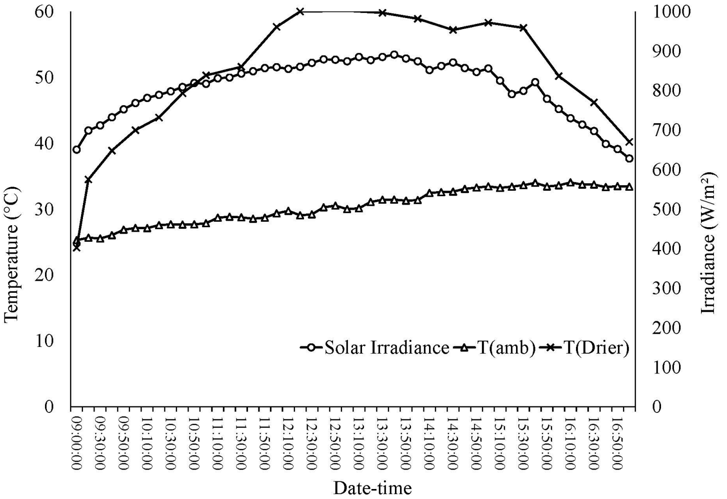

S 5: Solar irradiance, ambient temperature and drying chamber temperature, during solar processing of strawberry slices coated with F, M, G, A, X and un-coated R-R

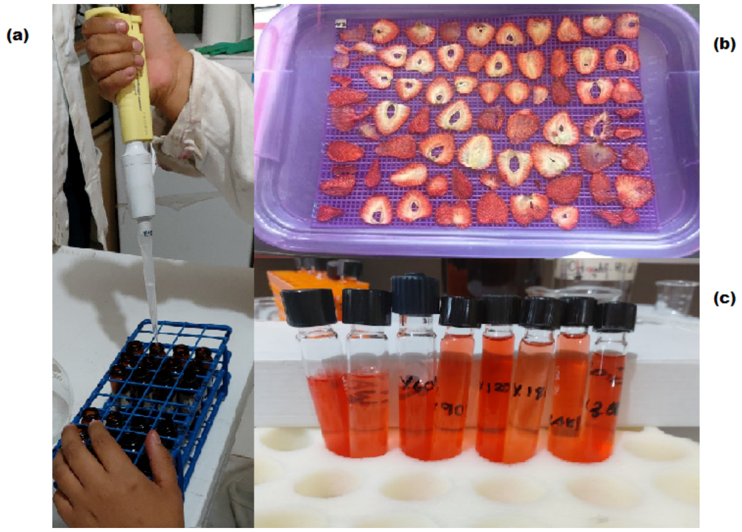

S 6: a) sample preparation, b) strawberry slices a) sample preparation, b) strawberry slices with different edible coatings during the solar drying; c) extracts (only for picture) from the slices in appropriate solvents for further storing under refrigeration in amber vials for TA and TPP assaying later on.

References

[1] A. Sharma, S. Gautam, and S. Wadhawan, "Xanthomonas," in *Encyclopedia of Food Microbiology (Second Edition)* (C. A. Batt and M. L. Tortorello, eds.), pp. 811 – 817, Oxford: Academic Press, second edition ed., 2014.

[2] D. Salarbashi, J. Bazeli, and E. Fahmideh-Rad, "Fenugreek seed gum: Biological properties, chemical modifications, and structural analysis– a review," *International Journal of Biological Macromolecules*, vol. 138, pp. 386 – 393, 2019.

[3] R. C. Garg, "Chapter 44 - fenugreek: Multiple health benefits," in *Nutraceuticals* (R. C. Gupta, ed.), pp. 599 – 617, Boston: Academic Press, 2016.

[4] A. A. Fadlelmoula, "22 - dietary gum arabic as animal feed additive," in *Gum Arabic* (A. A. Mariod, ed.), pp. 261 – 267, Academic Press, 2018.

[5] F. M. León-Martínez, L. L. Méndez-Lagunas, and J. Rodríguez-Ramírez, "Spray drying of nopal mucilage (Opuntia ficus-indica): Effects on powder properties and characterization," *Carbohydrate Polymers*, vol. 81, pp. 864–870, jul 2010.
